# CAR T Cell Therapy for Neuroblastoma

**DOI:** 10.3389/fimmu.2018.02380

**Published:** 2018-10-16

**Authors:** Rebecca M. Richards, Elena Sotillo, Robbie G. Majzner

**Affiliations:** ^1^Department of Pediatrics, Stanford University School of Medicine, Stanford, CA, United States; ^2^Stanford Cancer Institute, Stanford University School of Medicine, Stanford, CA, United States

**Keywords:** neuroblastoma, pediatric oncology, immunotherapy, CAR T cells, adoptive T cell therapy, clinical trials

## Abstract

Patients with high risk neuroblastoma have a poor prognosis and survivors are often left with debilitating long term sequelae from treatment. Even after integration of anti-GD2 monoclonal antibody therapy into standard, upftont protocols, 5-year overall survival rates are only about 50%. The success of anti-GD2 therapy has proven that immunotherapy can be effective in neuroblastoma. Adoptive transfer of chimeric antigen receptor (CAR) T cells has the potential to build on this success. In early phase clinical trials, CAR T cell therapy for neuroblastoma has proven safe and feasible, but significant barriers to efficacy remain. These include lack of T cell persistence and potency, difficulty in target identification, and an immunosuppressive tumor microenvironment. With recent advances in CAR T cell engineering, many of these issues are being addressed in the laboratory. In this review, we summarize the clinical trials that have been completed or are underway for CAR T cell therapy in neuroblastoma, discuss the conclusions and open questions derived from these trials, and consider potential strategies to improve CAR T cell therapy for patients with neuroblastoma.

## Introduction

Neuroblastoma is a tumor of childhood arising from neural crest cells. Often diagnosed during the first 10 years of life, it is the most common extracranial solid tumor in childhood and is responsible for 11% of pediatric cancer deaths in patients younger than 15 years of age (1). Approximately 650 patients are diagnosed in the United States with neuroblastoma each year, which accounts for 7.5% of all cancer diagnoses for children younger than 15 years old (2, 3). Clinical presentation and outcomes are extremely variable. Newborns and infants are often incidentally found to have adrenal tumors that spontaneously regress without therapy, while toddlers and older children frequently present with widely metastatic disease that requires multimodal intensive therapy including surgery, chemotherapy, radiotherapy, autologous stem cell transplant, differentiation therapy, and monoclonal antibody-based immunotherapy. Patients with localized disease typically have excellent outcomes, with >90% event free survival (EFS) rates 5 years after diagnosis (4). In contrast, patients with high risk disease (defined by age >18 months, extent of metastases, and histologic and genetic factors such as N-MYC amplification) historically have had poor long term survival prospects, with 5-year EFS of about 50% (5–8). Patients who do survive often suffer long term sequelae from their intense treatment including hearing loss, growth retardation, and secondary malignancies (9). This population therefore has a desperate need for novel therapies to improve survival and to decrease morbidity.

Antibody-based immunotherapy was recently integrated into frontline protocols for patients with high risk neuroblastoma. A pivotal phase III clinical trial published in 2010 revealed an increase in 2 year EFS from 46 to 66% and overall survival (OS) from 75 to 86% for patients who received adjuvant anti-GD2 monoclonal antibody given with IL-2, GM-CSF, and retinoic acid compared to patients who received retinoic acid alone (6). Incorporation of anti-GD2 monoclonal antibodies into therapy for neuroblastoma has been one of the most successful interventions to improve survival for high risk patients (6, 10–13). This success has firmly established a new paradigm for the treatment of neuroblastoma that includes immunotherapy.

While survival rates have improved since the adoption of anti-GD2 antibodies, ~50% of patients will relapse and eventually die from their disease (6). Additionally, 20% of patients are refractory to induction therapy at diagnosis and may not ever receive anti-GD2 antibody (14). These patients are in need of more potent and targeted approaches. One such approach is adoptive transfer of chimeric antigen receptor (CAR) T cells, which combine the specificity of an antibody with the cytolytic capacity of T cells in an MHC independent manner (15). CD19 and CD22 CAR T cells have demonstrated remarkable success in children with relapsed and refractory leukemia and lymphoma (16–20). While anti-GD2 monoclonal antibodies have been successful in treating patients with neuroblastoma metastases in their bone marrow, they have generally not been useful as single agents against bulky disease (21). CAR T cells have the potential for increased potency and durability compared to monoclonal antibodies and thus could overcome this challenge. Additionally, while antibodies generally do not penetrate the central nervous system (CNS) (22), CAR T cells are able to cross the blood-brain barrier (23, 24). Relapsed neuroblastoma of the CNS has emerged as a clinical entity since the adoption of anti-GD2 monoclonal antibodies, and CARs could present an answer to this challenging clinical problem (25, 26).

CAR T cells have already shown promise in clinical trials for neuroblastoma with several objective responses seen in early phase studies (27–31). In general, however, CAR T cell activity has not been as robust in neuroblastoma as in hematologic malignancies. There are many challenges in designing CAR T cells against neuroblastoma including suboptimal T cell persistence and potency (27–29), a paucity of tumor specific targets (32, 33), and an immunosuppressive tumor microenvironment (34, 35). However, CAR T cell engineering is accelerating at a rapid pace, with the aim to improve potency and specificity of tumor targeting (36–41). Neuroblastoma is an excellent testing ground for these new therapeutics since immunotherapy has already been validated for these patients. In this review, we will discuss the clinical experience to date with neuroblastoma-directed CAR T cells and the challenges of applying these powerful therapeutics to neuroblastoma patients. As CAR T cell design becomes more sophisticated, these agents are primed to become part of the multimodal approach used to treat patients with high risk neuroblastoma.

## Clinical experience

Much of the early clinical experience treating children with CAR T cells has been in hematologic malignancies, but neuroblastoma has also been an area of intense investigation, with a steady stream of clinical trials of CAR T cells for patients with relapsed or refractory disease since the early 2000s. Despite preclinical development of CAR T cells against a variety of neuroblastoma associated antigens, only those directed against GD2 and L1-CAM (CD171) have reached clinical trials. Table 1 summarizes completed and ongoing clinical trials.

**Table 1:** Summary of CAR T cell clinical trials for neuroblastoma.

**Clinical Trial**	**Study Design**	**Status**	**Target**	**scFv**	**Signaling domains**	**Response/toxicity**	**Location**	**References**
N/A	Phase I: N = 6	Completed	L1-CAM	CE7R	CD3ζ only	PR in 1/11 patients with limited disease burden, no DLT	Seattle Children's Hospital (Washington, USA)	(29)
NCT02311621	Phase I: N = 22	Recruiting	L1-CAM	CE7R	4-1BB.CD3ζ; CD28.4-1BB.CD3ζ	No objective responses, DLT with hyponatremia in two patients, self-limited rash in five patients	Seattle Children's Hospital (Washington, USA)	(42)
NCT00085930	Phase I: N = 19	Active, not recruiting	GD2	14g2a	CD3ζ only	CR in 3/19 patients, PR in 1/19 patients, response correlated with CAR T cell persistence, no DLT	Baylor College of Medicine/Texas Children's Hospital (Texas, USA)	(27); (28)
NCT01822652	Phase I: N = 11	Completed	GD2	14g2a	CD28.OX40.CD3ζ	No objective responses, no DLT	Baylor College of Medicine/Texas Children's Hospital (Texas, USA)	(43)
NCT02761915	Phase I: N = 12	Recruiting	GD2	KM8138	CD28.CD3ζ	Mixed response in 1/12 patients, no DLT	University College London (London, United Kingdom)	(30)
NCT02765243	Phase II: N = 34	Recruiting	GD2	Unknown	CD28.4-1BB.CD27.CD3ζ	PR in 15% of patients, no DLT	Zhujiang Hospital (Guangzhou, Guangdong, China	(31)
NCT03294954^*^	Phase I	Recruiting	GD2	14g2a	CD28.CD3ζ in invariant NKT cells	N/A	Baylor College of Medicine/Texas Children's Hospital (Texas, USA)	Unpublished
NCT02107963	Phase I	Completed	GD2	14g2a	OX40.CD28.CD3ζ	N/A	National Cancer Institute (Washington, D.C., USA)	Unpublished
NCT01460901	Phase I	Completed	GD2	14g2a	CD3ζ only	N/A	Children's Mercy Hospital Kansas City (Kansas, USA)	Unpublished
NCT03373097	Phase I/II	Recruiting	GD2	14g2a	CD28.4-1BB.CD3ζ	N/A	Bambino Gesu Hospital and Research Institute	Unpublished
NCT02919046	Phase I	Recruiting	GD2	14g2a	CD28.OX40.CD3ζ	N/A	Nanjing Children's Hospital (Nanjing, China)	Unpublished

This table summarizes the completed and ongoing clinical trials of CAR T cell therapy for neuroblastoma patients. Differences in the single chain variable fragments (scFv) and CAR signaling domains are highlighted, and clinical responses are summarized (trials for which no clinical data has been publicly presented are shaded in gray). NCT03294954 uses invariant NKT cells for CAR transduction, as opposed to T cells as in all other listed trials (

* *). Clinical references are provided. PR, partial response; CR, complete response; DLT, dose limiting toxicity*.

### GD2

The most-studied tumor associated antigen in neuroblastoma is GD2. GD2 is a disialoganglioside that is highly and nearly universally expressed on neuroblastoma tissue (44) and likely plays a role in tumor immune evasion (45). It is a natural choice as a target for CAR T cell therapy in neuroblastoma based on the success of anti-GD2 monoclonal antibody therapy (6, 10–12).

One of the first CAR T cells products tested in children was a first generation anti-GD2 CAR (containing only the CD3ζ endodomain but no costimulatory domain). In preclinical models, Rossig et al. demonstrated that GD2 was a viable CAR T cell target for neuroblastoma (46). To translate the preclinical promise of anti-GD2 CAR T cells into patients, Pule et al. aimed to treat patients in a manner that could enhance CAR persistence. CAR T cells with first generation signaling domains (CD3ζ only) had previously demonstrated limited persistence in human trials for other indications, indicating that the CD3ζ only intracellular domain was not sufficient for optimal activity (47–49). Rather than endowing the CAR with embedded costimulation, Pule and colleagues generated a T cell product that could receive physiologic costimulation through engagement of a native T cell receptor (TCR).

These researchers drew on experience from clinical trials in which Epstein Barr Virus-specific cytotoxic T lymphocytes (EBV-CTLs) were adoptively transferred to patients with EBV-associated malignancies (50–53). In those trials, T cell persistence of at least 3 months was seen even with relatively low doses of EBV-CTLs. Adding tumor specificity with a CAR construct was a logical next step to take advantage of the longevity of EBV-CTLs. A Phase I trial (NCT00085930) tested this approach by infusing EBV-CTLs co-expressing a first generation anti-GD2 CAR into relapsed and refractory neuroblastoma patients who were seropositive for EBV viral capsid antigen (27).

In this trial, EBV-specific lymphocytes were extracted from eleven patients with refractory or recurrent neuroblastoma, transduced with retrovirus encoding a GD2 CAR molecule (containing the single chain variable fragment (scFv) derived from Dinutuximab, 14g2a), and stimulated *ex vivo* with autologous EBV-transformed lymphoblastoid cell lines (LCLs). This product was called GD2 CAR-CTL. Concurrently, bulk T cells were transduced with the same GD2 CAR but activated through the native TCR with anti-CD3 antibodies (GD2 CAR-ATC). Each patient received between 2 × 10^7^ and 1 × 10^8^ cells/m^2^ of both GD2 CAR-CTL and GD2 CAR-ATC. A 12-base pair mutation between the receptor stop codon and the 3′ LTR allowed for comparison of *in vivo* durability of the two cell types by RT-PCR. There was little to no detection of GD2 CAR-ATCs after 2 weeks, but clear persistence of the EBV specific GD2 CAR-CTLs until on average 6 weeks, demonstrating that costimulation is vital for CAR T cell persistence. Four of the eight patients (50%) with evaluable tumors had a partial or complete response, though all later progressed. Responses included one patient with a complete response of an extradural parietal lesion as measured by MIBG, one patient with a complete response of extensive bone marrow disease, and two patients with significant tumor necrosis confirmed by imaging and biopsies. These data support the hypothesis that ongoing costimulation increases persistence *in vivo* and results in increased efficacy and durability of response. A subsequent study with longer follow up determined that even low levels of persistent cells correlated strongly with slower time to disease progression (28).

While using viral specific CTLs takes advantage of the native TCR machinery with physiologic stimulation, there is some evidence that co-engagement of a CAR and TCR can result in T cell exhaustion and decreased CAR persistence (54). Most CAR constructs now rely on embedded costimulation. The same group from Baylor produced a third generation CAR containing both the CD28 and OX40 costimulatory domains. Preclinical studies demonstrated that incorporation of tandem costimulation domains increased expansion of the engineered T cell product and augmented cytokine release (55, 56), which prompted testing this construct in clinical trials.

The third generation anti-GD2 CAR was administered to eleven patients with relapsed or refractory neuroblastoma. Patients were treated in one of three cohorts: GD2 CAR T cells alone, GD2 CAR T cells after lymphodepleting chemotherapy, or GD2 CAR T cells after lymphodepleting chemotherapy given with the PD-1 inhibitor pembrolizumab. Patients who received lymphodepletion with or without checkpoint blockade had increased expansion of their CAR T cells and longer CAR T cell persistence. Anti-PD-1 therapy did not appear to dramatically affect these parameters or efficacy. Unfortunately, even after patients received proper lymphodepletion, this CAR was found to have minimal activity with no measurable responses (43). One explanation for the lack of long-term persistence seen in this trial is tonic signaling of the CAR T cell caused by aggregation of the 14g2a anti-GD2 scFv, leading to T cell exhaustion and limited anti-tumor efficacy (57). T cell exhaustion, which will be further discussed below, has emerged as an important factor that can limit CAR efficacy and is highly dependent on costimulation molecules (57, 58).

Another Phase I trial of anti-GD2 CARs is underway in the United Kingdom (NCT02761915) utilizing an scFv based on a previously described humanized murine antibody KM8138 (59) that is fused to a CD28 costimulatory domain and CD3ζ. Based on promising preclinical data (60), this trial is enrolling children with relapsed or refractory neuroblastoma and evaluable disease in a dose escalation model. Preliminary results presented in abstract form demonstrate minor clinical response by imaging criteria and cytokine release syndrome (CRS) in at least one patient at higher dose levels, but CAR T cell persistence also appears to be limited (30). A fourth generation GD2 CAR (including CD28, 4-1BB, and CD27 costimulatory domains in addition to CD3ζ) is also being tested in a multi-institutional Chinese Phase II trial for high-risk neuroblastoma patients. An abstract presented in 2017 reported 15% of 34 patients with a partial response and no dose limiting toxicities. Two patients had significant tumor regression, one with two bulky lesions that regressed by >90% each and one with a reduction in retroperitoneal tumor dimensions and standardized uptake value (SUV) by PET scan measured 2 months after CAR T cell therapy (31).

Despite mixed results in the early GD2 CAR clinical trials, this target remains an area of intense focus. There are currently many ongoing preclinical studies focused on targeting GD2 as well as five open clinical trials of CAR T cells directed against GD2 for neuroblastoma patients (NCT03373097, NCT02761915, NCT02765243, NCT03294954, NCT02919046). While the experience thus far with GD2 CARs in clinical trials has established safety and feasibility, limited T cell persistence has emerged as a major hurdle to success.

### L1-CAM/CD171

Another target of interest in neuroblastoma is L1-CAM, an adhesion molecule that is overexpressed on neuroblastoma. Monoclonal antibody CE7 preferentially binds to a tumor-specific epitope of L1-CAM (61). The mechanism of tumor specificity has not been elucidated, but appears to be glycosylation-dependent (62–64). A first generation CAR containing the CE7 scFv, a CD4 transmembrane domain, and the CD3ζ intracellular signaling domain (CE7R CAR) demonstrated preclinical activity in xenograft models of neuroblastoma (65). A clinical construct was designed to include a selection-suicide fusion protein composed of hygromycin phosphotransferase and thymidine kinase (HyTK), allowing for CAR ablation with ganciclovir in the case of unforeseen toxicity. In a Phase I clinical trial of escalating doses of CE7R HyTK CD8+ CAR T cells, the authors demonstrated safety and observed no off-tumor, on-target toxicity. However, only one of six patients had a significant clinical response. That patient had limited disease burden, whereas the patients with higher disease burden had progressive disease. All patients ultimately died of their disease (29). Similar to GD2, lack of persistence of CAR T cells was also a major limiting factor in this study, which may have been related to the lack of costimulation in the CAR or to immunogenicity of the suicide HyTK protein (66).

To enhance the activity and persistence of L1-CAM directed CARs, the researchers then generated a second generation CAR (2G CE7 CAR) containing a 4-1BB costimulation domain and a truncated extracellular epidermal growth factor receptor (EGFRt) domain in place of the HyTK suicide switch (allowing for an alternative ablation strategy with cetuximab) (67, 68). Reassuringly, there was no significant clinical toxicity in non-human primates treated with 2G CE7 CAR T cells at doses 10–100 times higher than the doses employed in the clinical trial, though these primates did not have antigen positive malignancies (69).

A Phase I trial with the 2G CE7 CAR in rotation with a similar third generation product that also includes a CD28 endodomain is currently underway at Seattle Children's Hospital for recurrent or refractory high risk neuroblastoma patients (NCT02311621). Patients receive anti-L1-CAM CAR T cells in a defined ratio of 1:1 CD4:CD8 T cells. This strategy is based on previous successes of this controlled strategy for CAR T cell treatment of B-ALL and non-Hodgkin lymphoma at the same institution (70–72). Further study is required to determine the utility of a defined CD4:CD8 T cell product as this has not been tested in a randomized clinical trial, and equally impressive response rates have been obtained using non-selected populations of T cells or PBMCs after transduction (16, 17, 19, 20).

In a recently presented abstract, the researchers reported that L1-CAM CAR T cells infiltrate sites of disease in patients but appear to be causing off-tumor toxicity with transient skin rash (where the CAR T cells may colocalize with L1-CAM expressing normal cells) and poorly understood hyponatremia in some patients. Although these toxicities have all been transient and the trial is ongoing (42), the early finding of possible off-tumor, on-target toxicity is a reminder of the difficulty of identifying appropriate CAR-T cell targets (discussed further below).

## Challenges in targeting neuroblastoma with CAR T cells

Clinical experience thus far with CAR T cells for neuroblastoma indicates that T cell persistence is emerging as a major impediment for the success of these therapeutics. Outcomes have been encouraging but modest, with only a fraction of patients achieving measurable responses and very few patients demonstrating long term persistence of CAR T cells. In order to achieve the level of success that has been seen in hematologic malignancies, the field will have to address this challenge. Additionally, target selection is equally important, as many neuroblastoma targets are also expressed on normal tissues, creating the potential for off-tumor, on-target toxicity as may have been seen with L1-CAM CARs (albeit transiently). There may be a therapeutic window for CAR T cells against highly expressed tumor antigens that exhibit lower levels of expression on normal tissue, so this does not necessarily preclude these molecules as targets. Finally, as with other solid tumors, a complex, immunosuppressive microenvironment in neuroblastoma tumors presents a barrier for efficacious CAR T cell therapy.

### T cell persistence and exhaustion

CAR T cell persistence is essential for durable clinical responses (16, 47, 73–75). Long term follow-up of Baylor's first generation anti-GD2 CAR T cell trial demonstrated that time to disease progression was significantly delayed in patients whose T cells were detectable for longer (27, 28). In the trial of a first generation L1-CAM CAR, the only patient of six with a clinical response had detectable CAR T cells in the blood 56 days after treatment, while patients without objective response had shorter persistence (29).

CAR T cell persistence may be diminished due to T cell exhaustion. T cell exhaustion has primarily been studied in the setting of chronic antigen exposure including for viral infections (76, 77) and cancer (78–81). Exhausted T cells upregulate inhibitory receptors after excessive and continuous stimulation over a matter of days to weeks and exhibit diminished effector functions. T cell exhaustion appears to be partially reversible. This is fundamentally different from T cell senescence, which typically occurs over months to years, is associated with telomere shortening, and represents a terminally differentiated state without potential for reversibility or proliferation (82).

An exhausted CAR T cell phenotype has recently been described in GD2 CAR T cells, driven by antigen-independent tonic signaling (57). Long et al. explored why GD2 CAR T cells containing the 14g2a scFv appeared to be less functional than CD19 CAR T cells. The authors found that unlike the CD19 CAR, the GD2 CAR aggregated on the surface of T cells and subsequently triggered low level tonic signaling in the absence of antigen, which ultimately resulted in T cell exhaustion. Additionally, they demonstrated that integration of the CD28 costimulatory domain into tonically signaling CAR T cells amplified this phenotype, while inclusion of a 4-1BB costimulatory domain protected against T cell exhaustion (57). This finding is in line with clinical studies of CD19 CAR T cells, as those with 4-1BB costimulatory domains demonstrate long term persistence while those with CD28 costimulatory domains do not (16, 18, 19). Our group plans to open a clinical trial of GD2 CAR T cells with a 4-1BB costimulatory domain in early 2019, which will be the first such trial in North America.

Persistence can be affected by factors extrinsic to the CAR molecule. Early CAR T cell trials did not incorporate lymphodepletion prior to CAR T cell infusion, which may have compromised expansion of the engineered T cells (18, 43, 71). Lymphodepleting chemotherapy improves engraftment and efficacy and has become a standard part of CAR T cell regimens (83, 84). The mechanism of increased activity after lymphodepletion is thought to be depletion of regulatory immune cells and/or a reflexive increase in homeostatic cytokines IL-7 and IL-15 that drive CAR T cell proliferation (84–86). Given that endogenous cytokines may increase CAR efficacy, some groups have focused on increasing CAR potency by programming CAR T cells to secrete immunostimulatory cytokines locally (87, 88). Systemic infusion of cytokines is often associated with unacceptable toxicity (89–91), and overexpression of the cytokine receptor does not overcome a dearth of cytokines in the tumor microenvironment (92). Therefore, providing local and inducible cytokine release by the CAR T cells themselves is an attractive strategy. Initial reports have demonstrated improved potency of CD19 CAR when co-expressed with IL-7 (93), IL-12 (94), IL-15 (95), membrane bound chimeric IL-15 (88), and IL-21 (93). Further studies will be required to translate these results clinically and to see if this can be generalized to solid tumors and to neuroblastoma specifically.

Anti-carcinoembryonic antigen (CEA) CAR T cells were engineered to produce IL-12 only after engagement with target antigen by placing IL-12 under the control of a nuclear factor of activated T cells (NFAT) promoter. In a colon cancer model, CEA CAR T cells that expressed inducible IL-12 mediated greater tumor regression and abrogation of antigen negative tumor outgrowth. This effect was likely enhanced by activated macrophages that infiltrated the tumors in response to the locally secreted IL-12. (96). An alternative system combines oncolytic viruses that secrete cytokines IL-15 and CCL5 with anti-GD2 CAR T cell therapy in xenograft models of neuroblastoma in order to increase T cell infiltration and persistence (97).

Shum et al. recently described a system in which a constitutively active IL-7 receptor was co-expressed with a second generation GD2 CAR. This resulted in improved efficacy of GD2 CAR T cells *in vitro* and in a murine xenograft model of neuroblastoma (98). This modification did not lead to malignant transformation in short term assays, an important safety consideration as the IL-7 receptor was derived from a patient with T cell acute lymphoblastic leukemia (99). However, implementation of such a strategy into clinical trials will require caution due to the potential for delayed malignant transformation. These approaches to increase potency and persistence of CAR T cells are beginning to undergo testing in early clinical trials (NCT03635632), and may help to improve efficacy, durability, and ultimately clinical outcomes.

### Target selection and potential for toxicity

Choosing an optimal CAR T cell target in neuroblastoma and more generally in solid tumors is a daunting task. Much of the success of CD19 and CD22 CAR T cells hinges on the restriction of these targets to lymphoblasts and normal B cells, which are in large part dispensable with appropriate supportive measures (16–20). An ideal CAR target antigen is highly and homogeneously expressed on tumor cells with minimal expression on vital tissues. Fulfillment of these criteria is difficult for solid tumor antigens, as many antigens are expressed in cells of related origin.

Many antigens overexpressed on neuroblastoma are often present at lower levels in peripheral nerves and/or on other neural tissue (100–102), so an important consideration in the development of anti-GD2 CAR T cells is the potential for off-tumor, on-target toxicity. Anti-GD2 monoclonal antibodies cause pain requiring continuous infusion of narcotics for analgesia (103–106) due to their interaction with peripheral nerves and possibly engagement of the complement system (107). However, clinical trials of CAR T cells targeting GD2 have not resulted in toxicity despite clear signs of on-tumor efficacy (27, 28, 30, 43). Still, due to toxicity concerns, most anti-GD2 CAR T cells for clinical trials have been designed to include a “suicide switch” to allow for rapid ablation.

Oncologists have approached adoptive T cell therapy for solid tumors cautiously due to the overlap of antigen expression with normal tissues. There have been several incidents of off-tumor toxicity in human trials using engineered high affinity TCRs against MAGE-A3 (108) and MAGE-A12 (109) that cross reacted with normal tissue. Additionally, one patient with metastatic colon cancer died after treatment with a HER2-targeted CAR (110). The initial case report of this incident noted that there was pulmonary infiltration by CAR T cells that could be due off-tumor, on-target toxicity. However, that patient was administered a dose of CAR T cells that was found to be 100 times the maximum tolerated dose of CD19 CAR T cells as well as exogenous IL-2. She was found to have very high levels of circulating cytokines and our recent understanding of the toxicities associated with CAR T cells indicates that this was more likely to be caused by CRS than off-tumor, on-target toxicity (111). This is further supported by recent efforts at Baylor College of Medicine to target HER2 on pediatric sarcomas using CAR T cells. In carefully designed dose escalation trials conducted without and with lymphodepletion, anti-HER2 CAR T cells elicited no off-tumor, on-target toxicity but resulted in clinically significant responses including a complete response in a patient with metastatic rhabdomyosarcoma (112, 113).

One possible explanation for the lack of toxicity for both GD2 and HER2 CARs is the differential in antigen density between tumor cells and normal tissue. Antigen density is emerging as an important consideration for CAR efficacy. When our group engineered a CAR against Anaplastic Lymphoma Kinase (ALK) on neuroblastoma, there was a clear correlation between the number of surface molecules of target antigen and ALK CAR T cell efficacy. A threshold number of target molecules was required to elicit effector functions (114). *In vivo*, ALK CAR T cell efficacy was only seen when ALK expression was high on tumor cells. Similarly, in a Phase I trial of CD22 CAR T cells of children with ALL, after initially achieving a complete response, most patients relapsed with leukemia expressing lower levels of CD22 than their pre-treatment samples, apparently below the threshold for CAR efficacy (20). Others have found a similar relationship of CAR efficacy and antigen density in preclinical studies of CARs for targets including CD123, CD20, HER2, EGFR, and CD30 (115–121). This represents a paradigm shift in the field as it opens up potential therapeutic windows for targets expressed at low levels on normal tissue as long as expression on tumor is high (111).

As CARs are engineered to become more potent, they could also become more toxic due to recognition of lower levels of target. While several clinical trials of GD2 CAR T cells containing the 14g2a binder have been carried out without any reports of central or peripheral neurotoxicity (27, 28, 30, 43), one preclinical study of a high affinity GD2 CAR reported neurotoxicity and T cell infiltration in the brains of mice (122). However, studies of a CAR with the same high affinity binder in our laboratory do not cause neurotoxicity (123), calling into question whether the findings were truly due to off-tumor, on-target toxicity. The point remains, however, that as CAR T cells are better engineered to target low target antigen density tumor cells, there will be potential for increased toxicity and clinical trials must be conducted carefully.

### Novel targets

**Figure 1:**
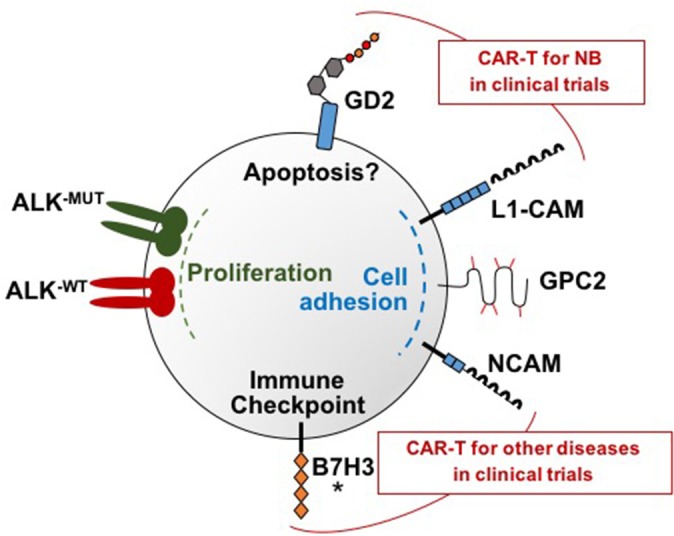
Molecular targets under investigation for CAR T cell therapy for neuroblastoma. There are a total of six neuroblastoma surface targets for which CAR T cells have been developed: GD2, L1-CAM, GPC2, B7H3, and ALK, and NCAM. These targets each have distinct functions that are depicted in this figure. Note that both wild type and mutated ALK are overexpressed on neuroblastoma samples and both can be targeted by the ALK CARs. GD2 and L1-CAM are the two targets currently in clinical trials for neuroblastoma. Clinical trials that include CAR T cells targeting NCAM are ongoing for multiple myeloma and AML but not yet for neuroblastoma. An asterisk marks B7H3 because clinical trials are currently being planned.

In addition to GD2 and L1-CAM, researchers are investigating several novel target antigens for CAR T cell therapy in neuroblastoma, and preclinical data are summarized below. Figure 1 depicts the targets currently under investigation for CAR T cell therapy for neuroblastoma.

#### Glypican 2 (GPC2)

GPC2 is a member of the glypican family of proteins (124), and is instrumental for growth and differentiation of axons in the developing nervous system (125, 126). Gene expression-based exploration of the surfaceome of neuroblastoma cells identified GPC2 as a cell surface molecule that is highly expressed in neuroblastoma with low expression on normal tissue, indicating that it may be an ideal candidate for CAR T cell based immunotherapy (32, 33). Retrospective review demonstrated significantly decreased survival in neuroblastoma patients with tumors expressing high levels of GPC2. Bosse et al. generated an anti-GPC2 antibody drug conjugate (ADC) that demonstrated strong antitumor activity in a patient derived xenograft (PDX) mouse model (32).

Concurrently, another group developed CARs containing heavy chain only scFvs against GPC2 with 4-1BB and CD3ζ endodomains. Anti-GPC2 CAR T cells demonstrated *in vitro* activity and *in vivo* clearance of human neuroblastoma xenografts (127). Though this study needs to be expanded to include a broader array of neuroblastoma cell lines and primary human samples, preliminary data suggest that GPC2 should be further evaluated as a clinical target for CAR T cell therapy in neuroblastoma. These studies also demonstrate the importance and power of a surfaceome approach to identify new targets for CAR T cell immunotherapy, with a <5-year turn-around time from target identification to development of therapeutics with potential for clinical application.

#### B7-H3/CD276

B7-H3 (CD276) is a checkpoint molecule expressed at high levels on many pediatric solid tumors including neuroblastoma (128–131). It plays a role in immune evasion (132) and metastatic potential (133), and overexpression correlates with poor prognosis in many cancers (134). These characteristics have made B7-H3 an attractive target for immunotherapeutic strategies, and early phase clinical trials with monoclonal antibodies have demonstrated encouraging results in both neuroblastoma and other malignancies (135–137). 8H9, a monoclonal antibody recognizing B7-H3, has been in clinical trials for more than 10 years; an 8H9 radioconjugate is an important element of a regimen for relapsed CNS neuroblastoma (NCT00089245) (135, 136). More recently, early phase clinical trials with a tumor specific anti-B7-H3 monoclonal antibody (MGA271) demonstrated safety and efficacy in adult malignancies (138). Our group has developed an active CAR targeting B7-H3 containing the scFv derived from MGA271 and efficacy is currently being explored in neuroblastoma (139, 140).

#### Anaplastic lymphoma kinase (ALK)

Several groups have identified anaplastic lymphoma kinase (ALK) as a potential oncogene in neuroblastoma (141–143). ALK is a receptor tyrosine kinase and, similar to GPC2, its expression is primarily restricted to the central and peripheral nervous system during fetal development (144). ALK regulates cell proliferation, differentiation, and apoptosis and has been implicated in many signaling pathways including PI3K/AKT, RAS/MAPK, and STAT3 (145). Activating mutations occur almost universally in familial neuroblastoma but also occur in a sizable percentage of sporadic neuroblastoma cases. Additionally, 15–20% of neuroblastoma patients overexpress wild type ALK in the absence of an activating mutation (146).

Anti-ALK CARs with a 4-1BB costimulatory domain were generated using previously described monoclonal ALK antibodies (147). ALK CAR T cells demonstrated *in vitro* activity but had limited efficacy *in vivo* in xenograft models of neuroblastoma (114). Investigations into the reasons for limited CAR efficacy demonstrated that ALK expression on the neuroblastoma cell lines used was below the threshold of antigen expression required for CAR activity. This finding demonstrates the importance of antigen density for CAR T cell efficacy (115–121).

#### Neural cell adhesion molecule (NCAM/cd56)

NCAM (CD56) is another glycoprotein that is important in neural development and is overexpressed on neuroblastoma (148). Similar to ALK and GPC2, it is overexpressed on tumors of neuroendocrine origin (149). It is also expressed on normal tissues, including most prominently on natural killer (NK) cells. Phase I and II clinical trials had demonstrated a favorable safety profile of anti-CD56 ADCs in solid tumors such as small cell lung cancer (150). The high and homogeneous expression on neuroblastoma and the limited toxicity of antibody-based therapy led one group to develop a CAR directed against CD56. This second generation CAR with a CD28 costimulation domain controlled tumor burden in a xenograft neuroblastoma model, but had only modest effects on survival (151). CD56 CAR T cells are being studied in clinical trials for relapsed multiple myeloma and for relapsed AML (NCT03473496, NCT03473457), though there are not yet published reports of any patient treated. Further investigation into CD56 as a target in neuroblastoma is warranted but off-tumor toxicity will need to be carefully monitored given significant normal tissue expression.

### Natural killer (NK) cell adoptive therapy

NK cells have long been recognized as important in neuroblastoma and killer cell immunoglobulin-like receptors (KIR) haplotypes are strongly correlated to survival (152–154). NK cells lack the specificity of T cells, but they have the capacity to kill infected and malignant cells without the prerequisite priming and sensitization to peptide-MHC complexes on the target cell surface. Instead, NK activity is regulated by a balance of activating and inhibitory receptors (155). Several trials are underway in which neuroblastoma patients receive adoptively transferred *ex vivo* expanded but unmanipulated NK cells (NCT02573896, NCT01857934, NCT02650648, NCT03209869).

Given their importance in control of neuroblastoma, researchers have attempted to augment the anti-tumor effects of NK cells in by imparting them with tumor antigen specific CARs. One group generated patient-derived NK cells expressing a second generation GD2-specific CAR, and demonstrated significant improvement in cytotoxicity against primary patient neuroblastoma cells compared to NK cells without a CAR (156). Similarly, expressing the GD2-CAR in an NK-92 cell line promoted *in vitro* cytotoxicity against neuroblastoma cell lines that were resistant to killing by the parental NK-92 cell line (157). NK cells do not have the same proliferative capacity as T cells, and clinical trials of adoptively transferred NK cells are often marked by short persistence and disappointing anti-tumor effect (158). The persistence of NK cells and invariant NK T cells can be increased by constitutive secretion of IL-15, an approach being studied in clinical trials for children with neuroblastoma at Baylor College of Medicine (NCT03294954) (159, 160).

### Engineering a successful CAR T cell product

In contrast to standard chemotherapy or “off the shelf” immunotherapies such as monoclonal antibodies, an important consideration for CAR T cell therapy is the ability to manufacture adequate quantities of a viable, maximally efficacious T cell product. Some patients have poor expansion and inadequate production of CAR T cells. One group hypothesized that myeloid derived suppressor cells (MDSC) in the apheresis product may interfere with T cell expansion, and found higher proportions of monocytes in PBMC concentrates to inversely correlate with fold expansion of CD19 and GD2 CAR T cells (161). CAR T cell quality is of particular concern for patients who have undergone chemotherapy, radiation, and/or stem cell transplant, all important elements of upfront neuroblastoma therapy. Data presented in abstract form describe T cell fitness in PBMC samples collected at diagnosis and after each cycle of chemotherapy from children with a wide variety of cancers including neuroblastoma. These data suggest that after chemotherapy, patients develop poor CAR T cell potential, defined by a low proportion of naïve T cells, mitochondrial dysfunction, and poor spare respiratory capacity (162). Further study is warranted to understand this phenomenon and whether it ultimately impacts CAR T cell efficacy in patients, as highly active CD19 CAR T cells have been successfully generated from most patients with heavily pretreated ALL (18, 19).

### Overcoming immunosuppressive tumor microenvironment (TME)

The immunosuppressive tumor microenvironment (TME) presents a significant barrier to successful CAR T cell therapy for neuroblastoma. Neuroblastoma tumors are intermixed with a suppressive cell population that includes tumor associated macrophages (TAMs) and regulatory T cells (Tregs). Presence of these cells predicts poor outcomes (34, 163). Tumors also express inhibitory ligands such as PD-L1 that dampen T cell responses (164–167). Furthermore, the TME contains an array of soluble factors such as TGF-β and IL-10 that act to directly inhibit T cells (34, 168–171). Finally, physical barriers such as stroma, extracellular matrix (ECM) and tumor associated vasculature prevent tumor infiltrating T cells from easily accessing their target (172–175).

#### Enhancing trafficking to neuroblastoma

For CAR T cell therapy of hematologic malignancies, the majority of malignant cells are located within the hematopoietic system. Solid tumors are not as readily accessible, a fact supported by data from early clinical trials in which GD2 CAR T cells were easily detectable in peripheral blood but rarely seen in post-treatment tumor biopsies (27). Optimal trafficking of T cells occurs when the effector T cells express a chemokine receptor that is complementary to chemokines that are rich in the tumor microenvironment, either excreted by tumor cells or surrounding tumor stroma. Expression of chemokine CCL2 has long been associated with more effective immune responses against neuroblastoma and it is secreted by neuroblastoma cell lines and primary tumor cells (176, 177). However, CAR T cells generated from neuroblastoma patients were found to have very low expression of the corresponding chemokine receptor, CCR2, despite expressing high levels of other chemokine receptors. Transgenic expression of CCR2b on GD2 CAR T cells in a neuroblastoma xenograft model improved kinetics of CAR T cell chemotaxis and greater anti-tumor efficacy (177).

#### Depleting suppressive immune cells

Assuming adoptively transferred T cells migrate appropriately to a solid tumor, they must circumvent many immunosuppressive factors within the TME. Many researchers are working to overcome this barrier. One strategy involves depleting suppressive immune cells. In a xenograft model of osteosarcoma, Long et al. observed that MDSCs decreased GD2 CAR T cell efficacy. When mice were treated with ATRA, which can induce differentiation of immature myeloid cells to a non-suppressive subtype (178), they had fewer suppressive MDSCs and there was a modest improvement in tumor control and survival (35). Alternatively, CARs themselves can be redirected against TAMs and regulatory T cells. One group took advantage of the dual specificity of CD123 CAR T cells against both Hodgkin lymphoma cells and TAMs. They found that with this strategy, they could target and eliminate TAMs and achieve durable remissions in Hodgkin lymphoma xenograft models (179).

#### Overcoming inhibitory signals

To evade the immune system, tumors express PD-L1, the ligand for PD-1, an inhibitory receptor on T cells. Engagement of this receptor dampens the native immune response (180) and blocking antibodies can “remove the brakes” and prompt an anti-tumor response, leading to success in early phase clinical trials (181–184). Neuroblastoma in particular was found to more frequently express PD-L1 than most other pediatric solid tumors. Additionally, PD-L1 expression [defined as >1% positive in tumor cells by immunohistochemistry, in line with some adult carcinoma scoring systems (185)] in neuroblastoma is associated with inferior survival (167).

PD-L1 upregulation on solid tumors can limit the efficacy of tumor-specific CAR T cells (186). Liu et al. postulated that they could improve anti-tumor control by combining CAR T cell therapy with a “switch-receptor” that would interrupt PD-1 inhibitory signaling. They endowed multiple CAR T cells with an additional chimeric receptor with a PD-1 extracellular domain directly connected to an intracellular CD28 co-receptor to provide costimulation and activation of T cells upon engagement with PD-L1. In all models, the switch receptor augmented CAR T cell function, and importantly, to a greater degree than anti-PD-1 monoclonal antibodies (39).

#### Interfering with inhibitory soluble factors

When neuroblastoma directed CAR T cells penetrate the suppressive immune milieu, they inevitably encounter suppressive factors including soluble cytokines that can suppress T cell function. These factors can be secreted by tumor cells or by surrounding stromal cells and include TGF-β, IL-10, galectin-1, and galectin-3 (34, 168–171); they represent potential targets to enhance CAR T cell efficacy. TGF-β in particular has importance in the neuroblastoma TME. Elevated levels of TGF-β transcripts in primary neuroblastoma samples were associated with shorter EFS (187), and blockade of TGF-β induced a more potent NK cell response in conjunction with anti-GD2 monoclonal antibody in a neuroblastoma xenograft model (188). T cells engineered to express dominant negative TGF-β receptors have been shown in a number of settings to improve efficacy of adoptive T cell therapy (189–191). This strategy was recently corroborated in a preclinical CAR model using an anti-prostate-specific membrane antigen (PSMA) CAR (192). PSMA CAR T cells coexpressed with the dominant negative receptor demonstrated increased proliferation, cytokine secretion, exhaustion resistance, persistence, and anti-tumor efficacy. With such pre-clinical promise, this construct has been incorporated into a clinical trial (NCT03089203).

#### Targeting tumor stroma

CAR T cells must penetrate physical barriers within the tumor stromal compartment that augment tumor growth and prevent infiltration of surveilling immune cells. Cancer associated fibroblasts (CAF) are the dominant cell type in the tumor stroma and express fibroblast activating protein-α (FAP) at high levels (173, 174, 193, 194). In a murine model of lung cancer, the efficacy of CAR T cells targeting the Ephrin Receptor tyrosine kinase EphA2 was enhanced by coadministration of anti FAP CAR T cells (195), providing proof of principle that anti-stromal CAR T cells can contribute to successful CAR T cell therapy in the solid tumor setting. Though this CAR has not yet been tested in neuroblastoma models, CAFs derived from primary neuroblastoma samples universally express FAP and enhance tumor engraftment and growth, and thus represent a potential target within the neuroblastoma TME (196).

T cell infiltration into tumors requires degradation of ECM proteins, including heparan sulfate proteoglycans (HSPG) (172). HSPGs are expressed on neuronal tissue during development and neuroblastoma cells are known to express some HSPGs at high levels (127). Activated T cells secrete heparanase to actively break down HSPG (197), but *ex vivo* culture of T cells causes downregulation of heparanase and abrogates their ability to degrade ECM (198). Expression of heparanase in a GD2 CAR T cell significantly improved tumor infiltration and antitumor activity in a neuroblastoma xenograft model (198), validating this as a potential method to improve CAR T cell therapy in stromal-rich tumors.

The immunosuppressive tumor vasculature presents a third physical barrier that may be a viable target to improve CAR T cell therapy. Vascular endothelial growth factor (VEGF) is a proangiogenic factor secreted by tumors, and can directly suppress immune cell infiltration of tumors (175). In a neuroblastoma xenograft model, anti-GD2 CAR T cells co-administered with the anti-VEGF antibody bevacizumab had superior anti-tumor activity over GD2 CAR T cells alone, thought to be primarily related to increased tumor infiltration by T cells (140).

## Conclusions

Immunotherapy with anti-GD2 antibodies has revolutionized the care of neuroblastoma patients, but there is still a great need for novel therapies for the patients with refractory or relapsed high risk disease. Early clinical trials with CAR T cells in neuroblastoma have demonstrated safety and shown some objective clinical responses. They have also provided insight into reasons for limited success, including lack of T cell persistence, difficulty in target antigen selection, and a suppressive tumor microenvironment. These challenges are universal in the CAR T cell field, in particular for solid tumors like neuroblastoma, and there are significant efforts underway to improve upon each of these domains. Successful CAR T cell therapy in neuroblastoma will require rational engineering approaches that address each of the above-mentioned barriers. Many studies presented in this review have encouraging pre-clinical results and thoughtful incorporation of some of these strategies into clinical trials will ultimately validate CAR T cells to treat neuroblastoma and improve patient outcomes.

## Author contributions

All authors listed have made a substantial, direct and intellectual contribution to the work, and approved it for publication.

### Conflict of interest statement

RM has a pending patent application for the use of GD2 CAR T cells in H3K27M mutant gliomas. The remaining authors declare that the research was conducted in the absence of any commercial or financial relationships that could be construed as a potential conflict of interest.
